# Sustainable Thermal Regulation of Electronics via Mitigated Supercooling of Porous Gallium‐Based Phase Change Materials

**DOI:** 10.1002/advs.202310185

**Published:** 2024-04-18

**Authors:** Seokkan Ki, Seongjong Shin, Sumin Cho, Soosik Bang, Dongwhi Choi, Youngsuk Nam

**Affiliations:** ^1^ Department of Mechanical Engineering Korea Advanced Institute of Science and Technology (KAIST) Daejeon 34 141 Republic of Korea; ^2^ Department of Mechanical Engineering Kyung Hee University Yongin 17 104 Republic of Korea

**Keywords:** gallium, liquid metal, phase change materials, supercooling, thermal regulation

## Abstract

Gallium liquid metal is one of the promising phase change materials for passive thermal management of electronics due to their high thermal conductivity and latent heat per volume. However, it suffers from severe supercooling, in which molten gallium does not return to solid due to the lack of nucleation. It may require 28.2 °C lower temperature than the original freezing point to address supercooling, leading to unstable thermal regulation performance along fluctuations of cooling condition. Here, gallium is infused into porous copper in an oxide‐free environment, forming intermetallic compound impurities at the interfaces to reduce the activation energy for heterogeneous nucleation. The porous‐shaped gallium provides ≈63% smaller supercooling than that of the bulk type due to large specific surface area (≈9,070 cm^2^ per cm^3^) and high wetting characteristics (≈16° of contact angle) on CuGa_2_ intermetallic layer. During repetitive heating‐cooling cycles, porous‐shaped gallium consistently shows propagation of crystallization at even near room temperature (≈25 °C) while maintaining stable performance as thermal buffer, whereas droplet‐shaped gallium is gradually degraded due to partial‐supercooled state. The findings will improve the responsive thermal regulation performance to relieve a rapid increase in temperature of semiconductors/batteries, and also have a potential for energy storage applications.

## Graphical abstract

Gallium liquid metal is a fascinating phase change material (PCM) due to its high thermal properties. However, supercooling restricts its suitability for thermal applications. Here, sustainable thermal regulation performance of gallium‐based PCM is demonstrated by increasing possibility of heterogeneous nucleation at the interface between a porous Cu structure and the infused gallium liquid metal.

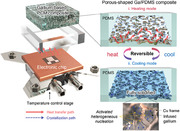

## 1 Introduction

Phase change materials (PCMs) have been investigated over several decades due to their high efficiency of thermal energy storage. Since PCMs can store a large amount of latent heat during solid‐liquid phase change, they have been widely applied to the systems including solar energy harvesting,^[^ ^1^ ^]^ air‐conditioning in buildings,^[^ ^2^ ^]^ and thermal management of batteries^[^ ^3^ ^]^ and electronics.^[^ ^4^ ^]^ In terms of the large scale (solar or building energy) applications, a total amount of energy conservation is more crucial compared to fast heat transport since the large volume of PCMs is available during the long daytime. Therefore, latent heat is the most concerning parameter among thermophysical properties. For transient cooling of electronics, however, thermal conductivity is also a significant factor for reducing a rapid increase in hot spot temperature. As electronic devices are prone to failure when subjected to drastic temperature fluctuations, introducing an effective thermal regulator with fast heat dissipation from hot spots to PCMs is essential.

PCMs are categorized into organics (e.g., paraffin), inorganics (e.g., salt hydrates, metals), and their eutectic mixture. Paraffin wax is the most common organic PCM due to its high latent heat (176–260 J g^−1^) as well as the cost‐effectiveness. However, it has low thermal conductivity (0.2–0.4 Wm^−1^K^−1^),^[^ ^5^ ^]^ leading to slow thermal response against the repetitive heat‐cool cycles. To improve the thermal conductivity of such type of PCMs, previous studies have incorporated conductive fillers (e.g., graphite,^[^ ^6^ ^]^ carbon nanotubes^[^ ^7^ ^]^) into the matrix. However, the obtained effective thermal conductivity (<10 Wm^−1^K^−1^) was still lower than that of metallic PCMs due to the constraint of volume fraction of the fillers. The latent heat per volume (≈200 J cm^−3^) is also lower than that of the metallic PCMs (≈1000 J cm^−3^),^[^ ^8^ ^]^ limiting a compact configuration of thermal packaging.

In contrast, metallic PCMs provide higher thermal conductivity with less performance degradation compared to that of organics, making them a promising candidate for effective thermal buffer. Several studies have investigated liquid metal (LM) PCMs as heat sink,^[^ ^9^, ^10^, ^11^, ^12^ ^]^ in which further extended protection time was attained compared to that of organics, presenting an improved thermal response. Although such LM PCMs provide superior thermal performance, there are still challenges to applying them into electronic packages due to their severe supercooling behavior. Supercooling means that a material does not return to the solid state even if the temperature drops below freezing point.^[^ ^13^ ^]^ When a molten PCM cools down, liquid‐solid phase change should occur, dissipating the stored latent heat to outside for reusability as thermal buffer. Otherwise, only sensible heat is stored during the heating period. Among the metallic PCMs, elemental Ga is one of the greatest candidates since it has a relatively high latent heat per weight (≈80 J g^−1^) and volume (≈488 J cm^−3^), suitable melting point (*T*_melt, Ga_ ≈29.8 °C) as well as high thermal conductivity (solid: ≈40.6 Wm^−1^K^−1^, liquid: ≈28.1 Wm^−1^K^−1^).^[^ ^14^ ^]^ However, molten Ga could sustain its supercooled state at sub‐zero temperature due to the polymorphism, where *α*‐Ga, *β*‐Ga, and other crystal structures coexist.^[^ ^15^ ^]^ From the view of homogeneous nucleation, *β*‐Ga has a further low freezing point (*T*_freeze_ = −16.25 °C) and is more favored to phase transition compared to *α*‐Ga that has a normal freezing point.^[^ ^16^ ^]^ Nano‐sized Ga droplets even maintain the liquid state below −100 °C due to the absence of nucleation for *α*‐Ga.^[^ ^15^ ^]^

To address the supercooling of Ga‐based LM, a few methods have been applied by inducing external energies such as mechanical vibration or electrical field in electrolyte solution.^[^ ^9^, ^17^ ^]^ In terms of active approach, placing a piece of solid Ga at 22 °C^[^ ^18^ ^]^ or touching the outside of Ga‐coated fiber below 29.8 °C^[^ ^19^ ^]^ was also investigated to instantly crystallized the supercooled liquid Ga. Although the immediate breaking of supercooling has been presented, these active strategies are unfavored for real‐world applications due to the increase in the system complexities. In contrast, passive strategies based on the activation of heterogeneous nucleation have also been investigated. Incorporating TeO_2_ seeds into Ga matrix for the activation of nucleation provided ≈43.7% mitigated supercooling (Δ*T*_super_ = *T*_melt_ – *T*_freeze_) compared to that of pristine Ga.^[^ ^20^ ^]^ But, the unstable freezing point was still shown as low as −13.8 °C. In addition, a graphene monolayer was deposited onto Ga frame for the catalytic effect, utilizing large area of preferential sites to stimulate heterogeneous nucleation.^[^ ^21^ ^]^ Graphene‐coated Ga provided ≈21.6% reduced supercooling (Δ*T*_super_ ≈17.1 °C) compared to bare Ga (Δ*T*_super_ ≈21.8 °C); but the temperature rise of Ga was confined up to 45 °C and the breaking of supercooling at near‐room temperature (>25 °C) was still difficult to be addressed. At room temperature, thin wires (Ga, Cu, Ni, Fe) with a diameter of 300 µm were investigated as nucleating agents.^[^ ^22^ ^]^ Among them, solid Ga wire demonstrated a reduction in supercooling of ≈77.7% compared to pristine Ga, owing to its similar crystal structure with liquid Ga, which promotes heterogeneous nucleation.

Encapsulation of Ga LM is another issue for thermal packaging of electronics. To avoid spilling out and short circuit when metallic PCMs become liquid state, previous studies have positioned bulk Ga into an external container^[^ ^9^ ^]^ or inner baseplate of the heat sink.^[^ ^12^ ^]^ For such configurations, however, additional thermal resistances between hot‐spots and PCMs are inevitable across the heat sink,^[^ ^23^ ^]^ envelop materials,^[^ ^24^ ^]^ and interfaces^[^ ^25^ ^]^ between the components. A few previous studies^[^ ^26^, ^27^, ^28^ ^]^ have fabricated LM droplets‐polymer matrix composites for electrical insulation, stretchability, as well as encapsulation. Although LM droplets have relatively larger interfacial sites for activation of heterogeneous nucleation compared to bulk type, they still present an irreversible phase change behavior against repetitive heat‐cool cycles. In fact, propagation of crystallization is impeded by amorphous gallium‐oxide (Ga_2_O_3_) layer between each shell of LM droplets,^[^ ^29^, ^30^ ^]^ leading to partially unsolved supercooling. Fast heat transport is also limited during the heating period due to the interfacial thermal resistances between each LM droplet.

To address the above‐mentioned challenges, we fabricated a porous, monolithic Ga liquid metal‐elastomer composite and applied it to electronics for demonstrating sustainable thermal regulation performance against the repetitive heat‐cool cycles. When infusing Ga into porous backbone structures, the interfacial thermal resistance between Ga and porous metal can be increased unless a sufficient contact interface is formed. Especially, an open‐cell Cu foam has an easily oxidized surface, leading to poor wetting characteristics even though it has a significantly high thermal conductivity (*k*_Cu_ ≈398 Wm^−1^K^−1^). We have utilized an oxide‐free infusing method, forming CuGa_2_ intermetallic compound (impurities) between interfaces. Both enhanced heat conduction and activation energy for heterogeneous nucleation were obtained by a scalable, one‐step process of Ga infusion. The introduced thermal buffer module alleviates a sharp increase in junction temperature while consistently addressing supercooling during repetitive thermal cycles. Moreover, for the type of porous Ga composite, a full propagation of crystallization was observed due to the monolithic network of Ga within the PCM composites.

## 2 Results and Discussion

### 2.1 Overall Description of the Introduced Thermal Regulation Module

**Figure 1:**
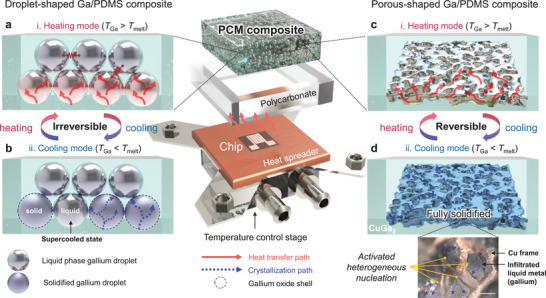
Introduced concept schematics of droplet‐ and porous‐shaped Ga/PDMS composites. a) Randomly arranged gallium droplets within polymer matrix are sequentially molten at heating mode (*T*_Ga_ > *T*_melt_). Thermal contact resistances exist along the heat transfer path between droplets, hindering the fast thermal responsiveness. b) At cooling mode (*T*_Ga_ < *T*_melt_), partially supercooled liquid gallium droplets are formed as propagation of crystallization is blocked by formation of gallium oxide shell (amorphous layer). c) The porous‐shaped Ga composite provides enhanced heat transfer performance due to the monolithically connected Cu/Ga structure. d) At relatively high temperature regime (> 20 °C), the porous‐shaped Ga composite recover the solid state, addressing the supercooling behavior by utilizing intermetallic compound (CuGa_2_) impurities between liquid metal and porous Cu structures. The scale bar of magnified image is 500 µm.

**Figure** **1** illustrates the concept schematic of the thermal regulation module consisting of the PCM composite with a mold, microheater chip, heat spreader, and a temperature control stage. The junction temperature (*T*_j_) of the microheater is regulated, absorbing the generated heat by Ga‐based PCM composite. Through the temperature control stage connected to a thermal bath, the overall temperature of the investigated module is managed. Considering previously reported Ga LM droplets‐elastomer composite,^[^ ^28^ ^]^ droplet‐shaped one has been determined as reference composite material in this study. Hence, we have investigated two types of PCM composites: i) droplet‐shaped and ii) porous‐shaped Ga/PDMS composite. For the former one, individual Ga droplets are randomly arranged within PDMS matrix (Figure 1a). During a heating mode (*T*_Ga_ > *T*_melt_), solid Ga sequentially melts from one droplet to another, forming heat conduction paths as themselves. However, interfacial thermal resistances still exist between droplets since they are encapsulated by nanoscale gallium oxide (Ga_2_O_3_) layer,^[^ ^31^ ^]^ which hinders fast heat transport. When a cooling mode begins (*T*_Ga_ < *T*_melt_), a few droplets are crystallized while the rest of them maintained their liquid state due to the supercooling of Ga (Figure 1b). Consequently, a partially supercooled state can persist unless a fresh heterogeneous nucleation occurs on each Ga droplet.

In contrast, the porous‐shaped Ga/PDMS composite provides advantages in both heating and cooling modes. Due to the monolithic morphology of the composite, a thermally conductive network of Ga can be established. Thus, a rapid heat absorption is offered during the heating mode (Figure 1c). This suggests that the regulation module can mitigate the sharp increase in junction temperature resulting from the heat generation of the processor chip. To overcome supercooling during the cooling mode, we intentionally build an interfacial CuGa_2_ layer between the monolithic Ga and supporting porous Cu backbone structure, avoiding the generated gallium oxides at the interface during fabrication. The chemically formed CuGa_2_ serves as impurities, increasing the possibility of heterogeneous nucleation within the expanded interfacial area along the porous surface. Following heterogeneous nucleation, the crystallization steadily propagates to the entire medium as the propagation path consists of pure Ga network, not blocked by amorphous oxide layer (Figure 1d).

### 2.2 Fabrication for Two Types of Ga‐Based PCM Composites

**Figure 2:**
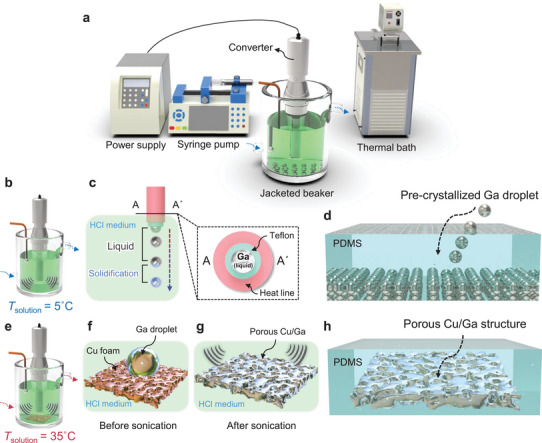
Fabrication procedure of droplets‐ and porous‐shaped Ga/PDMS composites. a) Overall schematic of fabrication setup. b) Liquid gallium is infused into the acidic medium by syringe pump. High‐frequency (20 kHz) ultrasonication is applied to the temperature‐controlled solution for immediately solidifying the gallium droplets. c) Syringe tube is wrapped by a heating line to prevent from clogging. d) Prepared gallium droplets are incorporated into PDMS matrix. e) To infiltrate liquid gallium into porous Cu, temperature of the solution increases over 35 °C. f) Due to the high surface tension of liquid metal, Ga maintains its puddle on Cu foam. g) Following high‐frequency sonication, Ga gradually spreads over the porous structure, forming intermetallic layer between interfaces. h) Prepared Cu/Ga foam is incorporated into PDMS matrix.

We prepared two types of Ga/PDMS composites by developing an experimental setup, where different procedures were applied for each case. **Figure** **2**a shows the schematic of the overall setup. Initially, to dissolve the pre‐formed gallium oxide layer, an acidic medium was contained in the jacketed beaker whose temperature was regulated by the connected thermal bath. Then, liquid Ga was infused into the acidic solution using a syringe pump to quantify the mass, applying ultrasonication for both cases. For the droplet‐shaped one, temperature of the solution (*T*_solution_) was maintained at 5 °C to immediately solidify the infused liquid Ga droplets. Although the solution temperature is maintained at ≈5 °C far below the freezing point of Ga (*T*_melt, Ga_ ≈29.8 °C), an instantaneous liquid‐solid phase change does not occur due to supercooling. To solve this issue, we applied high‐frequency ultrasonication (Figure 2b) to promote nucleation and immediate solidification, preventing Ga droplets from merging each other in the solution after injection. The syringe tube was covered by a heating line to suppress the clogging by the solidification of Ga during the injection (Figure 2c). The pre‐crystallized Ga droplets were gently dried by nitrogen blow and then, they were incorporated into PDMS matrix (Figure 2d). Although a volume expansion of ≈3.2% may occur when liquid Ga solidifies,^[^ ^32^ ^]^ there is no significant stress during repetitive heat‐cool cycles because pre‐solidified Ga droplets already occupy their maximum volume while PDMS curing. Since liquid Ga has a smaller volume compared to its solid phase, no additional stimulus (heterogeneous nucleation) is provided when the temperature decreases below the melting point. Here, the size of the droplets (*D*_drop_) can be varied along the fabricated conditions (Figure S1, Supporting Information).

For the porous‐shaped one, *T*_solution_ was elevated to 35 °C to maintain the liquid state of Ga (Figure 2e). Then, Cu foam was immersed into the acidic solution to eliminate the parasitic oxide layer. When Ga is injected into the porous Cu structure, it does not completely be absorbed due to the high surface tension (> 0.7 N m^−1^) of liquid Ga, presenting a puddle on the substrate (Figure 2f). To effectively laminate Ga to the porous Cu structure, we again applied high‐frequency ultrasonication, in which liquid Ga gradually cover the entire porous structure (Figure 2g), forming intermetallic compounds (CuGa_2_) as bonding layer at the interface between Ga and porous Cu. Then, the porous Cu/Ga composite was cured within PDMS matrix (Figure 2h). More detailed conditions of fabrication for Ga droplets and porous‐shaped Ga composite are provided in the Experimental Section.

### 2.3 Characteristics of Ga Droplets and Porous‐Shaped Cu/Ga Composite

**Figure 3:**
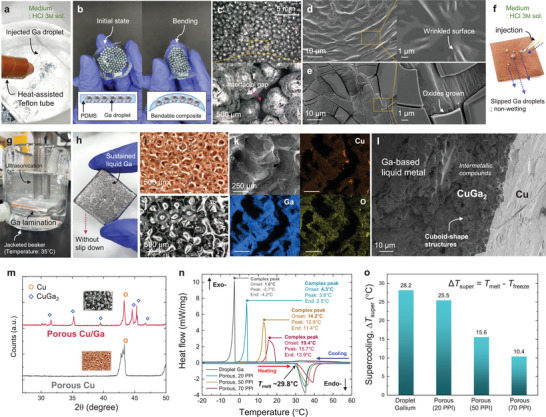
Characterizations of prepared gallium droplets and porous Cu/Ga composite. a) Automatically generated droplets by using heated syringe pump. b) Bending characteristics of droplet‐shaped Ga/PDMS composite. c) Optical images of gallium droplets. Magnified image shows that interfacial gap exists between droplets. d) SEM images of wrinkled gallium surface before harsh oxidation. e) After oxidation, ≈1 µm‐thick oxide layer is grown, which hinders fast heat transport and propagation of crystallization at heating and cooling mode, respectively. f) Gallium droplets slip down along the porous substrate in acidic medium. g) Gallium is gradually infiltrated into Cu foam by ultrasonication. h) Liquid gallium can be sustained within the porous structure. Optical images of porous foam i) before and j) after infiltration. k) EDS mapping results and l) cross‐sectional SEM image of porous Cu/Ga composite. m) XRD results for the demonstration of CuGa_2_ intermetallic compound. n) DSC results and o) the degree of supercooling for droplet‐ and porous‐shaped Ga composites.

**Figure** **3**a shows the automatically generated Ga droplets within the acidic medium. Separately dispersed droplets were obtained without merging each other in the solution. After incorporating into PDMS matrix, the droplet‐shaped one has a flexibility (Figure 3b), which is applicable to bendable electronic devices. Figure 3c shows the optical images of the fabricated Ga droplets. After drying, the gallium oxides formed again on the surface of the shell and interfacial gap between droplets exist. Figure 3d,e show scanning electron microscope (SEM) images of Ga surface before and after severe oxidation, respectively. Before harsh exposure to oxygen‐rich environment, only wrinkled surface (Figure 3d) is shown due to the native oxide nanofilm. However, ≈1 µm‐thick oxide layer is formed during the heating process (≈80 °C) as in Figure 3e, which may lead to high interfacial thermal resistance between Ga droplets. More importantly, the propagation of solidification can be interrupted by such oxide layer, leading to supercooling of droplet‐based one. The grown gallium oxide can serve as a passivation layer, thereby protecting the atomically smooth surface of liquid Ga from contacting external surfaces that promotes heterogeneous nucleation.^[^ ^30^ ^]^

Figure 3f shows the slipped Ga droplets onto the porous Cu substrate due to the non‐wetting characteristics in acidic medium. To laminate liquid Ga within the porous Cu backbone under the oxide‐free environment, ultrasonication was applied to the solution (Figure 3g). The porous structure has effectively retained the absorbed Ga without any loss, even in its liquid state as in Figure 3h. Regarding the flexibility of the porous Cu/Ga, we investigated the radius of curvature (*R*) along the thickness (0.2, 1.5, and 3 mm) of composites (Figure S2, Supporting Information). When the infused Ga is in a liquid state, all samples exhibited flexibility within the curvature range (*R* = 5.25 to 15.5 mm). Even in the solid state, the porous Cu/Ga composite with 0.2 mm and 1.5 mm of thickness showed bendability (*R* = 5.25 to 8.25 mm). Figure 3i,j show the optical images of the porous Cu/Ga before and after Ga absorption, respectively. We confirmed that all pores were thoroughly filled with Ga without air pockets for the case of high pore density (70 PPI). Higher magnified optical images including porous Cu/Ga having lower pore densities (20 and 50 PPI) are also provided in Figure S3 (Supporting Information). Energy dispersive X‐ray spectrometer (EDS) mapping analysis (Figure 3k) demonstrates that Ga LM was successfully laminated onto the porous Cu backbone. Chemically formed intermetallic compounds (CuGa_2_) were visualized by the cross‐sectional SEM image as in Figure 3l. A previous study also demonstrated that the intermetallic CuGa_2_ has been formed between Cu surface and eutectic GaIn alloy when both surface oxide layers are removed by sodium hydroxide aqueous solution.^[^ ^33^ ^]^ During high‐temperature annealing process (≈80 °C, 12 h), the cuboid‐shape structures were formed between Cu and liquid Ga. The existence of CuGa_2_ was further demonstrated by X‐ray diffraction (XRD) analysis (Figure 3m), in which the porous Cu/Ga provides several peaks related to CuGa_2_^[^ ^34^ ^]^ compared to that of pristine Cu foam.

To measure the degree of supercooling, we have observed phase change temperature of fabricated composites using differential scanning calorimetry (DSC) analysis. Figure 3n shows the results of DSC analysis for Ga droplet and porous Cu/Ga composites along the pore density (20, 50, and 70 PPI). All measured samples consistently presented endothermic peaks at ≈29.8 °C (*T*_melt, Ga_) during the heating period. However, as expected, the exothermic peaks were varied along the investigated Ga specimens due to the supercooling behavior. Among them, Ga droplet showed the lowest onset of solidification (*T*_freeze_ = 1.6 °C) due to the low probability of heterogeneous nucleation. On the other hand, the porous Cu/Ga provided higher onset of solidification (*T*_freeze_ ≈19.4 °C for 70 PPI) compared to that of the droplet‐based one. Latent heat is measured as ≈74.4 J g^−1^. The finding of particular significance is that pore density influences the degree of supercooling. Figure 3o shows the measured supercooling (Δ*T*_super_ = *T*_melt_ – *T*_freeze_) of the investigated samples. The highest supercooling (28.2 °C) was presented on Ga droplet while much lower values were shown for the case of porous‐shaped Ga composites. Here, ≈59.2% reduced supercooling was provided for the porous Cu/Ga composite with 70 PPI compared to that of 20 PPI. Based on the heterogeneous nucleation theory, we assumed that more available nucleation sites could be activated on the composite with higher pore density and larger interfacial area, which will be further discussed in Section 2.5.

### 2.4 Thermal Regulation Performance Along Heating‐Cooling Cycles

**Figure 4:**
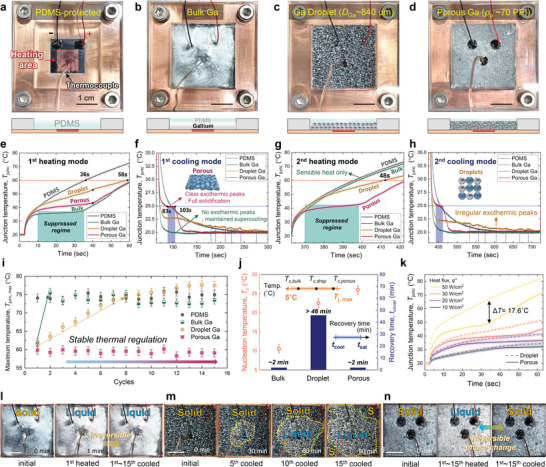
Experimental results of thermal regulation performance. Prepared test modules including a) PDMS‐protected, b) Bulk, c) Droplet‐, d) Porous‐shaped Ga/PDMS composites. Transient junction temperature of microheaters at e) the 1st heating mode, f) the 1st cooling mode, g) 2nd heating mode, and h) 2nd cooling mode. i) The maximum junction temperature (*T*_junc, max_) along the repetitive heat‐cool cycles. j) Nucleation temperature (*T*_c_) and recovery time (*t*_cover_) of bulk, droplet‐, and porous‐shaped Ga composites. k) Junction temperature variation along the applied heat flux. Top view of l) bulk type, m) droplet‐shaped, and n) porous‐shaped Ga/PDMS composites after heating or cooling cycles. Scale bars: 10 mm.

**Figure** **4**a‐d show the four representative thermal regulation modules: a) PDMS protection, b) bulk Ga, c) droplet‐shaped Ga (*D*_Ga_ ≈840 µm), and d) porous‐shaped Ga (*ρ*_pore_ = 70 PPI). For the PDMS‐protected module (Figure 4a), only sensible heat is stored during the heating mode, and other types of regulation modules (Figure 4b–d) can store both the sensible and latent heat, which may lead to further suppressed junction temperature of microheater. Here, the same amount of Ga (10 g) was used for Ga‐based modules (Figure 4b–d) to match the available latent heat of fusion (≈800 J). The specific preparation process of the thermal regulation modules and overall experimental setup were provided in Experimental Section (Figures S4 and S5, Supporting Information). In this analysis, heating (*q*′′= 40 W cm^−2^, *t*_heat_ = 1 min) and cooling (*T*_cool_ = 20 °C, *t*_cool_ = 5 min) conditions were fixed for the comparison of thermal regulation performance.

Figure 4e shows the increase in junction temperature (*T*_j_) at the 1st heating mode. As expected, the PDMS‐protected module showed the most rapid increase in *T*_j_, reaching 60 °C in only 36 s since only sensible heat (≈254 J) is stored from 20 to 60 °C. For the case of droplet‐shaped one, reduced trend of *T*_j_ was provided, showing extended protection time (58 s for 60 °C). Most of the Ga droplets were melt, absorbing both sensible (148 J) and latent heat (800 J). However, the slope of increase in *T*_j_ was not significantly reduced since the interfacial thermal resistance between Ga droplets led to the slow thermal response. Meanwhile, the porous‐shaped one showed further suppressed trend of *T*_j_, providing 43.5 °C at 40 s, which is ≈18.2% lower *T*_j_ compared to that of the droplet‐shaped one (53.2 °C). Due to the monolithic Ga within the connected Cu backbone, the improved heat conduction promoted fast solid‐liquid phase change of Ga. The most reduced *T*_j_ was observed for bulk‐type Ga, providing distinctly suppressed regime (10–35 s at 36 °C). After the entire medium of bulk Ga was melt (≈35 s), however, *T*_j_ was sharply increased to 60 °C as the latent heat was fully consumed. Considering the significantly suppressed *T*_j_, it seems that the bulk Ga has the highest thermal regulation performance at the 1st heating mode. Such a flat stage of junction temperature is attributed to time‐dependent variation in heat dissipation. From the onset of the heating until ≈35 s, the heat dissipation per unit area of the microheater steadily increases as a result of the solid‐liquid phase change heat absorption from the Ga‐PCM. In fact, after liquid fraction of Ga reached 1, ≈50% reduced heat dissipation on microheater was confirmed by numerical simulation. The effect of latent heat absorption on bulk Ga compared to PDMS‐protected module is provided in Figure S6 (Supporting Information).

At the 1st cooling mode (Figure 4f), the investigated modules presented different trend of *T*_j_ along the cooling time. PDMS‐protected module showed no specific exothermic peaks because the stored thermal energy was dissipated by only sensible heat without phase change. It also has the longest recovery time (*t*_cover_ > 150 s) to 20 °C (*T*_cool_) due to the low thermal conductivity of PDMS (0.15 Wm^−1^K^−1^). For the bulk Ga, there are no exothermic peaks along the overall cooling period, which means that the molten Ga by the 1st heating does not return to solid phase even at 20 °C due to the supercooling (*T*_melt, Ga_ = 29.8 °C). In the case of droplet‐shaped Ga, it is observed that the exothermic peaks are randomly distributed along the cooling time (vertical blue dot lines). We confirmed that heterogeneous nucleation was activated at about 22.5 °C due to the higher possibility of activation by the enlarged surface area of Ga droplets compared to that of bulk Ga. However, as the droplets have not monolithically connected each other, the propagation of crystallization is interrupted, resulting in a partial‐supercooled state as described in Figure 1b. On the other hand, the porous‐shaped one showed a clear exothermic regime where the onset of nucleation point was observed at ≈25 °C. The stored latent heat was dissipated at once only in ≈20 s, and no further exothermic peaks were observed since all liquid Ga returned to solid state, which indicates the full solidification in a short time.

At 2nd heating mode (Figure 4g), we have observed the different trend of *T*_j_ for Ga LM‐based composites compared to that of the 1st heating mode. The PDMS‐protected module showed an identical result compared to that of the 1st heating as it only utilized sensible heat during the cycles. For the bulk Ga, however, a significantly degraded cooling capability was measured, whose thermal regulation performance is even similar to that of PDMS‐protected one (see grey and green line). Since the stored latent heat still keep in the liquid‐state Ga after the 1st cooling, available thermal storage is only sensible heat during 2nd heating. The droplet‐shaped Ga also showed reduced thermal regulation performance since the available latent heat was reduced by partially supercooled Ga droplets. In fact, the protection time for reaching 60 °C of *T*_j_ was shortened from 58 s to 48 s due to the reduced latent heat. In contrast, the porous‐shaped Ga showed stable thermal regulation performance, providing a clear suppressed regime due to the fully solidified state of Ga after the 1st cooling. Even after 2nd cooling mode (Figure 4h), the bulk‐type Ga still remained fully supercooled state, by which we can expect that thermal storage performance at 3rd heating mode will be identical. The droplet‐shaped one also showed irregular exothermic peaks due to the locally addressed supercooling of Ga droplets. On the other hand, the porous‐shaped Ga exhibited a consistent solidification behavior, preparing for the subsequent heating mode.

To examine the stability of thermal regulation performance, we conducted 15 cycles of heating‐cooling experiments (Figure 4i). Here, the maximum junction temperature (*T*_j, max_) is defined as the highest *T*_j_ measured at each cycle. First, PDMS‐protected one provided consistent *T*_j, max_ (≈74.5 °C) as this module utilized sensible heat only. For the bulk Ga, although the low *T*_j, max_ (≈62 °C) was measured at the 1st cycle, significantly degraded *T*_j, max_ (≈75 °C) were repeatedly observed during the remained cycles from 2^nd^ to 15^th^. This is because the supercooling was not addressed even after 15 repeated cooling periods as in Figure 4l. One of the interesting results is that the *T*_j, max_ trend of the droplet‐shaped Ga. Along the number of cycles, the *T*_j, max_ gradually increased from ≈61.5 °C (initial cycle) to ≈77.5 °C (15th cycle), presenting a significant thermal degradation (16 °C). As in Figure 4m, the supercooled area within the overall Ga droplets gradually expands, resulting in the reduced thermal regulation performance. Meanwhile, the porous‐shaped Ga provided the highest thermal stability, presenting only 0.5 °C of standard deviation in *T*_j, max_ within total 15 cycles. This is attributed to the occurrence of liquid‐solid phase change during every cooling mode, breaking the supercooling. No partial‐supercooled state was observed during overall cycles as in Figure 4n. To further demonstrate its practical ability as a thermal buffer, a hundred heating‐cooling cycles were conducted, during which the stability of the thermal regulation performance was also consistently maintained. (Figure S7, Supporting Information.)

For high responsive thermal regulation of electronics, a recovery time for liquid‐solid phase change is also crucial parameter. Figure 4j shows both the nucleation temperature (*T*_c_) and recovery time (*t*_cover_) along the morphology of Ga‐based composite. Here, *T*_c_ was measured by tracking initial exothermic peak along the cooling from *T*_j, max_ to 5 °C for each case. In addition, *t*_cover_ was defined as the time from the beginning of the cooling mode (*t*_cool_) to reaching the saturation temperature (*t*_sat_ = 5 °C). For example, *t*_cover_ of bulk Ga was only ≈2 min because an initially activated nucleation could be easily propagated to the entire medium of pure Ga LM. However, *T*_c_ was observed at about 10.5 °C due to the severe supercooling, which means that a high cooling energy is required for addressing the supercooling of bulk Ga. Although the droplet‐shaped Ga can obtain a higher *T*_c_ (22.5 °C) compared to that of bulk Ga, too long *t*_cover_ (> 46 min) was measured because all the partially supercooled Ga droplets should be solidified. Since numerous Ga droplets individually exist within PDMS matrix, the propagation of crystallization is blocked, presenting irregular exothermic peaks during the cooling mode. For the porous‐shaped Ga composite, however, ≈26 °C of *T*_c_ was shown due to the improved possibility of heterogeneous nucleation by the interface between Cu foam and laminated Ga LM. In addition, as this composite is monolithically connected, a short *t*_cover_ (≈2 min) was obtained, providing a full propagation of solidification.

Figure 4k shows the *T*_j_ trend along the time when we applied the different heat loads (10 to 50 W cm^−2^) to the thermal regulation modules. Here, the dot‐line and solid line indicate the *T*_j_ of droplet‐shaped and porous‐shaped Ga, respectively. For the case of low heat flux (10 W cm^−2^), the difference in *T*_j, max_ was only ≈2.3 °C as small amount of latent heat was consumed for both cases. This could be explained by the sustained suppressed regime without sharp increase in *T*_j_ during the overall heating period. As the heat flux increases, however, the difference in thermally suppressed regime becomes clear between each module. For example, at 30 W cm^−2^ (orange line), the porous‐shaped Ga provided the suppressed regime over 50 s while the droplet‐shaped one showed gradually increased *T*_j_, reaching 51.5 °C of *T*_j, max_. When the heat flux became 50 W cm^−2^ (yellow line), the latent heat of porous‐shaped Ga was fully consumed by the enhanced heat conduction path within the monolithic Cu/Ga structure, showing different trend of *T*_j_ from ≈34 s. During the heating mode of 1 min, the maximum difference between *T*_j, max_ of each case was over 17 °C, highlighting an importance of the fast thermal response. The improved thermal responsiveness and transient solid‐liquid phase change phenomena within Ga composites at 40 W cm^−2^ were also demonstrated by simplified numerical simulations, which are provided in Figure S8 (Supporting Information).

### 2.5 In‐Depth Analysis for Supercooling

**Figure 5:**
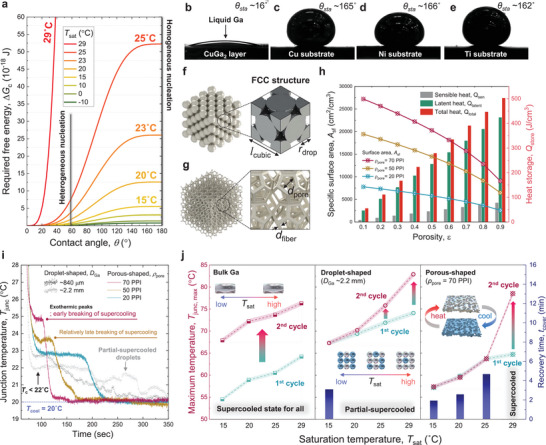
Analysis of heterogeneous nucleation and supercooling behavior. a) Required free energy along the equilibrium contact angle (*θ*) and saturation temperature (*T*_sat_) of gallium. Static contact angle (*θ*_sta_) of gallium droplet on b) CuGa_2_, c) Cu, d) Ni, and e) Ti substrates. Schematics of f) face centered cubic (FCC) and g) porous structures for calculating specific surface area of droplet‐ and porous‐shaped Ga/PDMS composites, respectively. h) Calculated specific surface area (*A*_sf_) and heat storage (*Q*_store_) along the porosity and pore density of composites. i) Transient exothermic peaks for analyzing heterogeneous nucleation along the diameter of gallium droplets and pore density of porous‐shaped Ga/PDMS composites. j) The maximum junction temperature (*T*_junc max_) and supercooling behavior along the saturation temperature (*T*_sat_) for various types of Ga/PDMS composites.

To further explain the reduced supercooling of the porous‐shaped Ga composites, we have calculated the nucleation energy barrier using the classical theory of heterogeneous nucleation^[^ ^35^ ^]^:

(1) $$\Delta G_{c}=\frac{16\pi \gamma_{LS}^{3}T_{melt}^{2}}{3\Delta H_{p}^{2}{\left(T_{m}-T_{sat}\right)}^{2}}\;\left(\frac{2-3\cos\;\theta +\mathrm{co}s^{3}\theta }{4}\right)$$

where Δ*G*_c_ is the free energy required for heterogeneous nucleation, γ_*LS*_ is the interface energy of liquid‐solid Ga (55.9 mJ m^−2^), *T*_m_ is the melting point of Ga (29.8 °C), Δ*H_p_* is volume fusion enthalpy (473.6 MJ m^−3^), and *θ* is the equilibrium contact angle.^[^ ^13^ ^]^ **Figure** **5**a shows the required free energy (Δ*G*_c_) versus equilibrium contact angle (*θ*) along the saturation temperature (*T*_sat_). Based on this calculation, the highest free energy was required at 180° regardless of *T*_sat_. Since homogeneous nucleation (*θ* = 180°) occurs away from the surface of the system, the onset of nucleation is much more difficult compared to that of heterogeneous nucleation. As the *θ* decreases to 0°, Δ*G*_c_ sharply reduces, indicating a higher likelihood of the onset of heterogeneous nucleation. To estimate the required free energy, we have measured static contact angles (*θ*_sta_) of liquid Ga on various substrates (CuGa_2_, Cu, Ni, and Ti) in acidic environment. The lowest *θ*_sta_ (≈16°) was measured on CuGa_2_ layer (Figure 5b), while high static contact angles over 160° were observed on the other substrates (Cu, Ni, and Ti) as in Figure 5c–e. It means that a high possibility of activation of heterogeneous nucleation on CuGa_2_ layer is expected compared to other substrates. For example, based on the trend of Δ*G*_c_ curve at 25 °C of *T*_sat_, ≈6.4 times lower energy is required for activation of nucleation when *θ* has been reduced from 180° to 60°.

Since such heterogeneous nucleation and crystallization occurred at the interface between mating materials, an interfacial area is also a critical parameter for breaking supercooling of the investigated Ga‐based composites. To estimate the total interfacial area of Ga droplets within PDMS matrix, we have calculated specific surface area (*A*_sf_) of spherical droplets in a defined unit cell (FCC; face‐centered cubic). Here, we assumed that the arranged droplets have the highest packing factor as in Figure 5f. As the side length (*l*_cubic_) of a unit cell is calculated as $l_{cubic}=\;2r_{drop}\sqrt{2}$, the atomic packing factor (APF) is ≈0.74 based on following equation:

(2) $$APF\;=\frac{N_{drop}\cdot V_{drop}}{V_{unit\;cell}}\;=\frac{4\cdot \frac{4}{3}\pi r_{drop}^{3}}{l_{cubic}^{3}}\;$$

When *r*_drop_ (radius of Ga droplet) is ≈420 µm, the number of droplets (*N*_drop_) per unit volume (1 cm^3^) is ≈2386. Then, total specific surface area (*A*_sf_) is calculated as ≈53 cm^2^ per cm^3^. As the *r*_drop_ become reduced, *A*_sf_ can be further expanded; however, supercooling would be more intense due to the lack of path for propagation of crystallization as discussed in Figure 4m.

Meanwhile, to obtain the specific surface area (*A*_sf_) of a porous medium, we have calculated correlations based on a previous literature^[^ ^36^ ^]^:

(3) $$A_{sf}=\frac{3\pi d_{fiber}\left[1-e^{\left\{\left(1-\varepsilon \right)/0.04\right\}}\right]}{{\left(0.59d_{pore}\right)}^{2}}\;$$

(4) $$d_{pore}=0.0254/\rho_{pore}\;$$

(5) $$d_{fiber}=\frac{d_{pore}\cdot 1.18\sqrt{\left(1-\varepsilon \right)/\left(3\pi \right)}}{\left[1-exp\left\{\left(\varepsilon -1\right)/0.04\right\}\right]}\;$$

where *d*_pore_ is effective diameter of a pore, *d*_fiber_ is a fiber diameter, *ε* is porosity of the porous medium (Figure 5g). Figure 5h shows the calculated *A*_sf_ and available heat storage (*Q*_store_) per unit volume (1 cm^3^) along the porosity (*ε*). As can be seen, a higher *A*_sf_ can be achieved by either increasing pore density (*ρ*_pore_, from 20 to 70 PPI) or reducing porosity (*ε*, from 0.9 to 0.1). In fact, the porous medium can have approximately 3 times expanded *A*_sf_ when porosity becomes reduced from 0.9 to 0.1 at a fixed pore density (70 PPI). However, if a low‐porosity Cu foam is applied, the available heat storage becomes significantly reduced. When porosity decreased from 0.9 to 0.1, over 447 J of *Q*_total_ is out of use due to the large portion of latent heat (84.4%) compared to the sensible heat (15.6%) within total heat storage (*Q*_total_). The high‐porosity (0.9) Cu foam already shows the superior specific surface area (*A*_sf_ ≈9070 cm^2^ per cm^3^) compared to that of droplet‐based one (≈53 cm^2^ per cm^3^), the possibility of breaking supercooling may grow up. To achieve a similar level of *A*_sf_ as a porous medium, the droplet‐based one needs to have a diameter (*D*_drop_) of at least 10 µm or less (Figure S9, Supporting Information). For example, ≈9067 cm^2^ per cm^3^ of *A*_sf_ can be obtained from Ga droplet that has 4.9 µm of *D*_drop_. In terms of thermal behavior, however, the freezing temperature (*T*_freeze_) was measured as low as −24.5 °C even for droplet‐based composite that has over 10 µm of *D*_drop_. It means that supercooling become more severe when *D*_drop_ reduced to 4.9 µm, leading to increased thermal resistance between droplets during heating mode and unavailable latent heat after cooling. As a result, we hypothesize that the porous‐shaped Ga composite with a high pore density (*ρ*_pore_, 70 PPI) and porosity (*ε*, 0.9) can simultaneously minimize supercooling behavior and maximize heat storage capacity. This is especially relevant for a low saturation temperature (*T*_sat_) due to the need for low free energy (Δ*G*_c_) and the expanded interfacial area between liquid Ga and the CuGa_2_‐based supporting backbone.

To demonstrate the above‐mentioned assumptions, we have observed the exothermic peaks of thermal regulation modules including droplets‐ (*D*_Ga_ = 840 µm, 2.2 mm) and porous‐shaped (*ρ*_pore_ = 20, 50, and 70 PPI) Ga composites. Figure 5i shows the variation of *T*_j_ during cooling mode (*T*_cool_ = 20 °C, *t*_cool_ = 5 min) after initial heating mode (*q*′′= 40 W cm^−2^, *t*_heat_ = 1 min). First, the porous‐shaped one with a high pore density (70 PPI) showed clear exothermic peaks near 25 °C as discussed in Figure 4f. With a lower pore density (50 PPI), however, exothermic peaks occurred at lower temperature regime (≈23.5 °C) compared to that of 70 PPI, indicating a relatively late breaking of supercooling. Considering the case of the lowest pore density (20 PPI), we confirmed that the specific surface area (*A*_sf_) related to pore density affects the supercooling, in which large *A*_sf_ leads to early breaking of supercooling due to the expanded nucleation sites between liquid Ga and CuGa_2_ substrates. We can also observe that all the porous‐based regulation modules have provided a clear regime of exothermic peaks because a continuous propagation of crystallization is possible by monolithically connected Ga regardless of pore density. These trends of supercooling are also corresponding to the results of DSC curve provided in Figure 3n. For the case of droplet‐shaped composites, a regulation module with a larger diameter (*D*_Ga_ ≈2.2 mm) was compared to previously investigated one (*D*_Ga_ ≈840 µm). As diameter of Ga droplets increased to ≈2.2 mm, ≈62% decreased *A*_sf_ was calculated (from 53 to 20.2 cm^2^ per cm^3^). As can be seen in light grey dot‐lines of Figure 5i, the module with a large *D*_Ga_ showed a more reduced nucleation temperature (*T*_c_ < 22 °C) compared to the previous one (*T*_c_ > 22.6 °C). After nucleation point, the module also showed irregular exothermic peaks within the overall cooling mode due to the discontinuity of Ga LM droplets integrated in PDMS matrix. Considering the low specific surface area (*A*_sf_ ≈ 20.2 cm^2^ per cm^3^) and separately distributed Ga droplets (*D*_Ga_ ≈2.2 mm), severe supercooling is inevitable. We can easily expect that thermal storage using latent heat may become reduced for the following heating period.

Finally, to investigate the effects of saturation temperature (*T*_sat_) along the morphology of Ga/PDMS composite, heat‐cool cycles were carried out based on the specific experimental conditions (*q*′′= 40 W cm^−2^, *t*_heat_ = 1 min, *t*_cool_ = 5 min, *T*_cool_ = 15–29 °C). Here, the maximum junction temperature (*T*_j, max_) and recovery time (*t*_cover_) were measured along the *T*_sat_ (Figure 5j). For the case of bulk Ga, the supercooled state has been maintained at the overall range of *T*_sat_ (15 to 29 °C) due to the relatively high free energy required and lack of activated nucleation sites at the interface compared to that of other types of Ga composite. Since all samples remained in a liquid state without returning to a solid state, there was no *t*_cover_. Therefore, thermal regulation performance was significantly degraded on the 2nd heating cycle, leading to 12–13.4 °C higher *T*_j, max_ compared to the 1st heating. Different trends on breaking of supercooling were shown for the case of droplet‐shaped composite. At 15 °C of *T*_sat_, all the liquid metal droplets (*D*_Ga_ ≈2.2 mm) returned to be solid Ga (*t*_cover_ > 3 min), presenting identical *T*_j, max_ (≈67.3 °C) within the 1st and 2nd heat exposure. As *T*_sat_ increased, however, partial‐supercooled Ga droplets were shown as inset schematic in Figure 5j. About 83 °C of *T*_j, max_ was presented after cooling at 29 °C, which is ≈11.9% higher than that of the 1st cycle. As discussed in Figure 5a, the required free energy for nucleation increases at high *T*_sat_, resulting in only a few droplets breaking supercooling. Therefore, there is no *t*_cover_ for all cooling temperature range except for 15 °C of *T*_sat_. Meanwhile, the porous‐shaped one (*ρ*_pore_ = 70 PPI) showed more reduced supercooling compared to bulk or droplet‐shaped Ga composites. At 15 °C of *T*_sat_, ≈2 min of *t*_cover_ was consumed, which is ≈1.6 times faster than that of droplet‐shaped one. Due to the advantage of the propagation of solidification through the monolithic Ga structure, *t*_cover_ is much shorter than droplet‐shaped module where each Ga droplet should be individually solidified. Even near‐room temperature range (20–25 °C), the heterogeneous nucleation could be activated at the interface between liquid Ga and CuGa_2_ layer due to both low static contact angle (*θ*_sta_ ≈16°) and high specific surface area (*A*_sf_ ≈9070 cm^2^ per cm^3^) of porous‐shaped composite (*ρ*_pore_ = 70 PPI). The *t*_cover_ at 25 °C of *T*_sat_ was even lower than 5 min, which again underlines the advantage of propagation of solidification. Until *T*_sat_ reached 25 °C, *T*_j, max_ was consistent between the 1st and 2nd cycles because of the fully addressed supercooling during each cooling mode.

## 3 Conclusions

In summary, we report a porous‐shaped Ga/PDMS composite to obtain both fast heat absorption performance and reduced supercooling behavior of Ga‐based PCM by infusing Ga LM into the open‐cell Cu foam. Compared to the droplet‐shaped Ga composite, the network of porous‐shaped one not only improves heat conduction path for alleviating junction temperature, but also reduces degree of supercooling to obtain sustainable thermal regulation performance. Due to the impurities (CuGa_2_ intermetallic compounds) between Ga LM and Cu foam, heterogeneous nucleation is easily activated at the interfaces, in which a larger specific surface area led to more reduced supercooling because of the high possibility of crystallization. In fact, DSC analysis clarified that supercooling reduced over 59% when the specific surface area of Cu foam increased from 2591 cm^2^ per cm^3^ (20 PPI) to 9069 cm^2^ per cm^3^ (70 PPI). Based on the developed thermal regulation module under 40 W cm^−2^ of heating condition, porous‐shaped Ga provided ≈18.2% lower junction temperature than that of droplet‐shaped one, presenting a clear *T*_j_ suppressed regime and exothermic peaks for heating and cooling mode, respectively. In addition, the porous‐shaped Ga have presented stable thermal regulation performance over a hundred heat‐cool cycles, repetitively breaking supercooling at 20 °C of *T*_sat_. Due to the low required free energy for heterogeneous nucleation and large surface area at the interface, the porous‐shaped Ga composite has addressed supercooling even at 25 °C while other types of Ga‐based PCM maintained their supercooled state. We believe that our findings can pave the way for high‐performance passive thermal management of real‐world applications including electronics, batteries, and data centers.

## 4 Experimental Section

### Preparation of Gallium Droplets

First, 3 mL of liquid gallium (Ga metal 99.99+, HKK solution) was filled into a syringe (10 mL, KOVAX). The length of syringe tip was extended by Teflon tube (DuPont). Then, outer wall of the tube was wrapped by a heating line to prevent solidification of liquid Ga during injection. Using a syringe pump (Fusion 100, Chemyx), liquid Ga was injected into the hydrochloric acid solution (HCl 3 mol L^−1^, SAMCHUN Chemicals) at 0.5 mL min^−1^ of flow rate. Temperature of the solution (*T*_solution_) was maintained at 5 °C by jacketed beaker connected to refrigerated circulating bath (LC‐LT212, LK Lab). During injection, ultrasonication (40–50% Amp. 20 kHz) was applied to the solution by a tip‐type sonicator (VCX‐750, SONICS). Fabricated Ga droplets were dried by nitrogen blow for 30 min at 25 °C to evaporate the residue of solution. The diameter of Ga droplets was controlled by varying conditions of injection (Table S1, Supporting Information).

### Fabrication of Porous‐Shaped Copper/Gallium

Based on the identical setup as mentioned above, temperature of the acidic solution was increased up to 35 °C. Copper foam (Cu metal 99.9, American Elements) was cleaned in an ultrasonic bath with acetone for 10 min at room temperature, then rinsed with ethanol and de‐ionized water. Cleaned foam with various pore densities was first immersed into the acidic solution to exfoliate the parasitic oxide layer, and then 10 g of liquid Ga was infused onto the porous Cu substrate. Here, high‐power ultrasonication (40‐50% Amp. 20 kHz) was applied to the solution for an effective infiltration of liquid Ga into open‐cell Cu foam. To evaporate residue of the solution on the surface after absorption process, the porous Cu/Ga composite was dried at 70 °C for 30 min.

### Characterizations

The surface morphologies of gallium‐based materials were observed using an optical microscope (VHX‐950F, KEYENCE) and scanning electron microscope (SEM) (SU‐8230, Hitachi). Elemental compositions of the porous Cu/Ga were investigated by energy‐dispersive X‐ray spectroscopy (EDS) connected to SEM. Existence of CuGa_2_ intermetallic compounds within Ga‐infused porous Cu was demonstrated using X‐ray diffractometer (XRD) (D8 Advance, BRUKER). Phase change temperatures with endo‐ and exothermic peaks of droplet‐ and porous‐shaped Ga were investigated by modulated differential scanning calorimeter (MDSC) (204 F1 Phoenix, NETZSCH) at the KAIST Analysis Center for Research Analysis (KARA). For analysis, heating and cooling rate is set as 10 °C min^−1^ from −50 °C to 60 °C. Specimens were prepared by cutting Cu foam into a shape of crucible pan, then infusing gallium liquid metal.

### Fabrication of Pt‐Based Microheater

To imitate the heat‐generating processor chip, microheaters were fabricated (Figure S10, Supporting Information). First, 1 µm of silicon dioxide (SiO_2_) insulation layer was deposited on silicon (Si) wafer (525 µm, P‐type, i‐Nexus) using a plasma‐enhanced chemical vapor deposition (PECVD) method before coating of photoresist (KMPR‐1000, KAYAKU). The photoresist (PR) was spin‐coated and lithographically patterned into a square serpentine shape. 20 nm of titanium (Ti) adhesion layer and a 200 nm of platinum (Pt) layer is subsequently deposited through the electron‐beam evaporation process. The patterned photoresist layer covered with Ti/Pt layer was removed by lift‐off method. Target heating area (1 cm^2^) and heat flux range (up to 50 W cm^−2^) were designed by adjusting thickness, width, and number of turns of Pt‐resistive layer. A calibrated thermocouple (K‐type, Omega) was attached to the top surface of the heating area using a thermally conductive adhesive (OB‐200, Omega). Approximately 100 µm of PDMS layer was coated onto the microheater for electrical insulation. The prepared microheater was attached to the copper heat spreader using a commercial thermal interface material (8.5 Wm^−1^K^−1^, MX‐4).

### Preparation of Thermal Regulation Modules

Test modules were prepared to evaluate thermal regulation performance of developed Ga‐based composites. Polydimethylsiloxane (PDMS) elastomer base and curing agent (Sylgard 184, Dow Corning) were mixed at a 10:1 mass ratio using a planetary mixer (ARE‐310, THINKY). For the PDMS‐protected module (Figure 4a), ≈4.3 g of PDMS is filled into the regulation module to assume that only sensible heat is available. For the bulk Ga‐based module (Figure 4b), 10 g of Ga and 0.17 g of PDMS were sequentially filled into the PCM volume. Droplet‐shaped and porous‐shaped Ga/PDMS modules were also prepared by pouring ≈0.17 g of PDMS onto Ga droplets and porous Cu/Ga composite, respectively. Detailed procedures for preparation of the porous‐shaped Ga/PDMS module are explained in Figure S4 (Supporting Information). In addition, the specific materials, dimensions, and thermal properties of the regulation module are summarized in Table S2 (Supporting Information).

### Overall Test Platform and Procedure

The prepared modules were mounted on the temperature control stage where the conditions were converted between the cooling and insulation. As in Figure S5a,b (Supporting Information), the stage was installed onto the optical table (vibration isolation, DAEIL Systems) to minimize vibration effect that may induce the unexpected breaking of supercooling during the cooling mode. The thermal bath is connected to the stage to control the conditions including coolant temperature (*T*_cool_), and flow rate. Flow rate and pressure drop are measured by flowmeter (F‐1000, Blue‐White) and pressure transducer (SIG, Sensys) in the cooling loop, respectively. The heat flux of microheater varies by DC power supply (E3634a, Keysight). The junction temperature of heater (*T*_j_) was recorded by DAQ data acquisition device (NI‐9213, National Instruments) connected to a data logging program (LabVIEW, National Instruments). The top view of the solid‐liquid phase change phenomena was visualized by a CMOS camera. First, temperature of the module was maintained at investigated cooling temperature (5–29 °C) to initialize the condition by coolant circulation, in which all Ga remained solid‐state. Before heating mode, the liquid cooling was paused, and air was injected by opening the ball valve (Figure S5c, Supporting Information). Here, the forced convection heat transfer is excluded, and the bottom of the module is insulated. In this way, most of the generated heat can be transferred to the Cu heat spreader and PCM composite. For heating mode, heat is applied to the microheater for 1 min, in which solid Ga gradually melted to the liquid phase, consuming the latent heat of fusion. After heating mode (voltage off), the valve was closed, and then the water coolant was circulated again to cool down the temperature of the module (cooling mode). The coolant temperature was varied (*T*_cool_ = 5–29 °C) since the breaking of supercooling is significantly related to the temperature swing (Δ*T*_swing_ = *T*_melt_ – *T*_cool_) of the Ga‐based PCM. During repetitive heating‐cooling cycles, the transient junction temperature (*T*_j_) was tracked to analyze both thermal regulation performance and supercooling behavior (Figure S5d, Supporting Information).

### Thermal Modeling and Analysis

To numerically elaborate the thermal buffer during the heating mode, the transient and 3D finite element method (FEM) simulation was conducted using computer‐aided engineering (CAE) software (COMSOL Multiphysics). For the heat transfer calculation, a time‐dependent energy equation based on Fourier's law of heat conduction is calculated as follows:

(6) $$\rho C_{p}\;\frac{\partial T}{\partial t}=\;\nabla \left(k\nabla T\right)$$

where *ρ*, *C*_p_, and *k* indicate the material's density, heat capacity, and thermal conductivity, respectively. Here, the heat capacity (*C*_p_) term is divided into the sensible and latent heat capacity as follows:

(7) $$C_{p}=\theta_{s}\;C_{p,s}+\theta_{l}C_{p,l}+L_{s\to l}\frac{\partial \alpha_{m}}{\partial t}$$

(8) $$\alpha_{m}=\frac{1}{2}\;\frac{\theta_{l}-\theta_{s}}{\theta_{s}+\theta_{l}}$$

where *θ_s_* (solid) and *θ_l_* (liquid) is the mass fraction of each phase (*θ_s_* + *θ_l_* = 1). *L*_s→_ *_l_* is latent heat of fusion from solid to liquid phase (80 J g^−1^). Transition interval during the solid‐liquid phase change is set as 5 °C referring to the previous study^[^ ^12^ ^]^ around the melting point of Ga (*T*_melt_ = 29.76 °C). The thermal conductivity (*k* = *θ_s_ k_s_* + *θ_l_ k_l_*) and density (*ρ* = *θ_s_ ρ_s_* + *θ_l_ ρ_l_*) are also varied considering the mass fraction of each phase during the phase change. A quarter‐schematic of the simulation platform is provided in Figure S11 (Supporting Information). A boundary heat source is provided on the top surface of Pt‐based heating area. The natural convective heat transfer coefficient (*h*_nat_ = 5 W m^2^ K^−1^) is applied to the top and side surface of the module. Since the shape of the module is symmetrical, the symmetry conditions are applied to sides of the module for reducing the computational cost. Convergence criterion is set as 1×10^−6^ with the time step of 1s to investigate the temperature and liquid fraction along the time variation.

### Contact Angle Measurement

To quantify the wetting characteristics of Ga onto various substrates (CuGa_2_, Cu, Ni, and Ti), the static contact angle (*θ*_sta_) of Ga was measured using an automatic measurement system (Smart Drop, Femtobiomed). 10 µL of Ga droplet was dispensed using a micro‐pipette in an acidic environment (immersed in HCl 1 mol L^−1^ solution). Here, CuGa_2_ surface was prepared by contacting liquid Ga with Cu surface, rinsing with acetone and isopropyl alcohol (IPA), and then storing it in an incubator at 35 °C. Then, residual Ga on the CuGa_2_ surface was removed, followed by another round of rinsing with acetone and IPA.

## Conflict of Interest

The authors declare no conflict of interest.

## Supporting information

Supporting Information

## Data Availability

The data that support the findings of this study are available from the corresponding author upon reasonable request.
