# Supplementary material for: Sustainable Thermal Regulation of Electronics via Mitigated Supercooling of Porous Gallium‐Based Phase Change Materials
Source: Adv Sci (Weinh). 2024 Apr 18;11(23):2310185. doi: 10.1002/advs.202310185 (PMC11186057; doi:10.1002/advs.202310185)
Supplement: Supplementary file 1 — Supporting Information [file ADVS-11-2310185-s001.pdf]

ADVANCED SCIENCE
Open Access

Supporting Information

for *Adv. Sci.*, DOI 10.1002/advs.202310185

Sustainable Thermal Regulation of Electronics via Mitigated Supercooling of Porous Gallium-Based Phase Change Materials

*Seokkan Ki, Seongjong Shin, Sumin Cho, Soosik Bang, Dongwhi Choi and Youngsuk Nam**

Supporting Information

Sustainable Thermal Regulation of Electronics via Mitigated Supercooling of Porous Gallium-Based Phase Change Materials

*Seokkan Ki, Seongjong Shin, Sumin Cho, Soosik Bang, Dongwhi Choi, and Youngsuk Nam**
*ysnam1@kaist.ac.kr

Contents

1. Optical images of the prepared Ga droplets with various diameters (Figure S1)
2. Bendability of the porous Cu/Ga composite with various thicknesses (Figure S2)
3. Microscopic images of porous Cu/Ga composite with various pore densities (Figure S3)
4. Preparation process of thermal regulation module (Figure S4)
5. Experimental setup and procedure of thermal regulation module (Figure S5)
6. Numerical results of junction temperature, heat dissipation, and liquid fraction along the time at heating mode for PDMS-protected and bulk Ga-based regulation modules. (Figure S6)
7. Stability of the porous-shaped Ga/PDMS composite over 100 heat-cool cycles (Figure S7)
8. Numerical results of temperature and liquid fraction of Ga-PCM along the time (Figure S8)
9. Comparison of specific surface area between droplet- and porous-shaped Ga/PDMS composites (Figure S9)
10. Fabrication process of Pt-based microheaters (Figure S10)
11. A quarter-schematic of the simulation model (Figure S11)
12. Conditions for injection of liquid Ga for various droplet size (Table S1)
13. Characteristics of the investigated thermal regulation module. (Table S2)

1. Optical images of the prepared Ga droplets with various diameters (Figure S1)

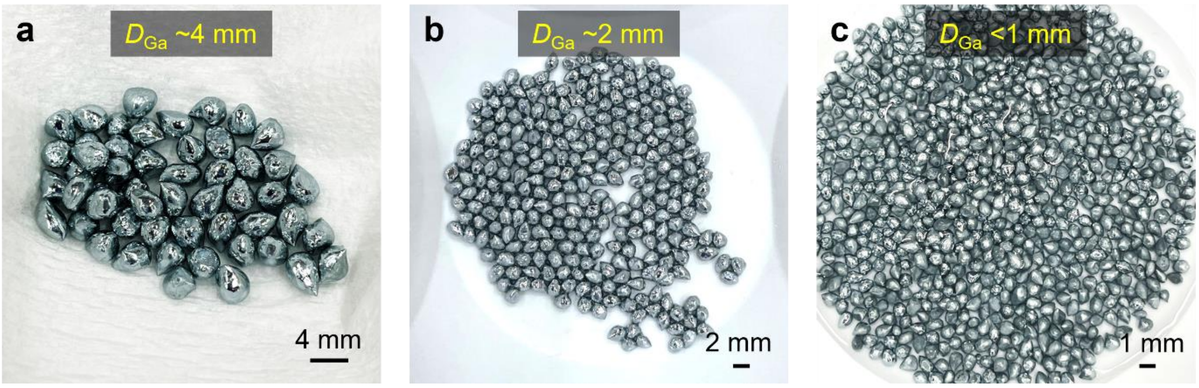

Figure S1. Optical images of the prepared Ga droplets with various diameters (D_{Ga}). a) ~4 mm, b) ~2 mm, and c) below 1 mm of diameter, respectively.

2. Bendability of the porous Cu/Ga composite with various thicknesses (Figure S2)

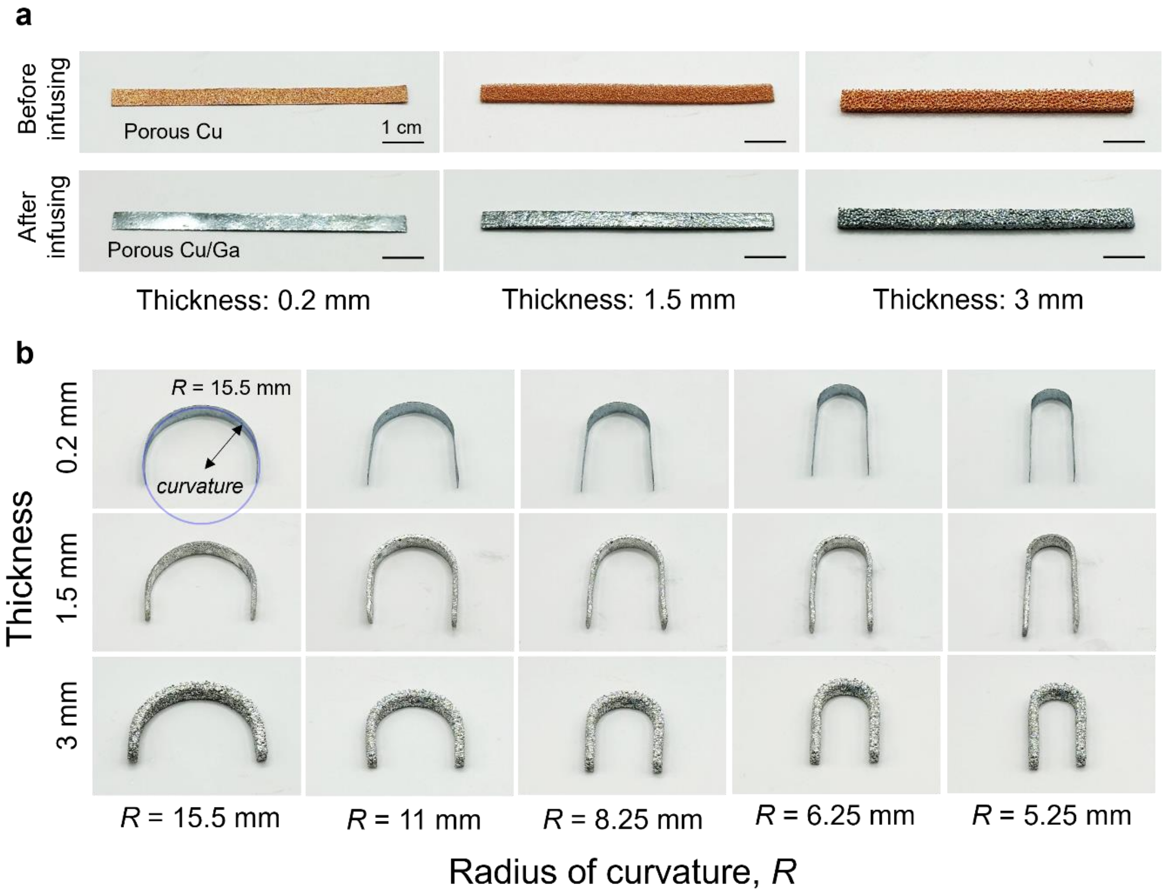

Figure S2. a) Porous Cu (above) and the porous Cu/Ga composite (below) along the various thicknesses. Scale bars: 1 cm for all images. b) flexible Cu/Ga foam along the investigated thickness (0.2 mm, 1.5 mm, and 3 mm) and radius of curvature (R = 5.25 to 15.5 mm).

3. Microscopic images of porous Cu/Ga composite with various pore densities (Figure S3)

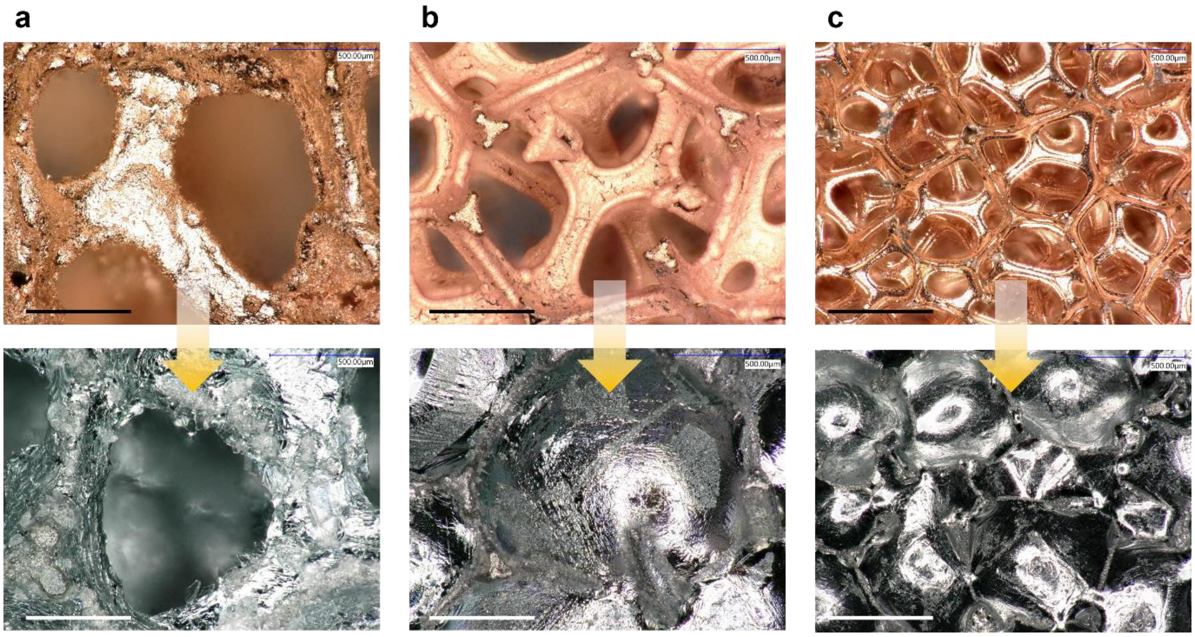

Figure S3. Microscopic images of porous Cu/Ga composite with various pore densities. a) 20 PPI, b) 50 PPI, and c) 70 PPI, respectively. Scale bars: 500 µm for all images.

4. Preparation process of thermal regulation module (Figure S4)

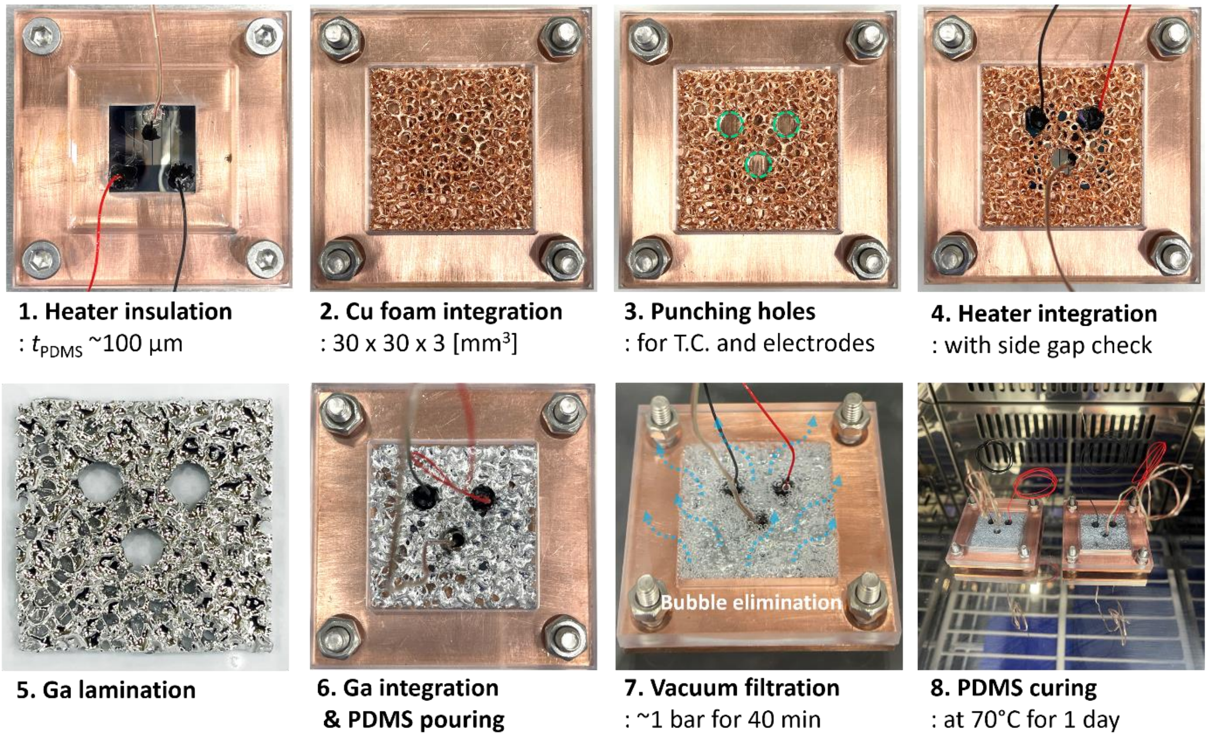

Figure S4. Preparation process of thermal regulation module. Procedure details are provided beneath each image.

5. Experimental setup and procedure of thermal regulation module (Figure S5)

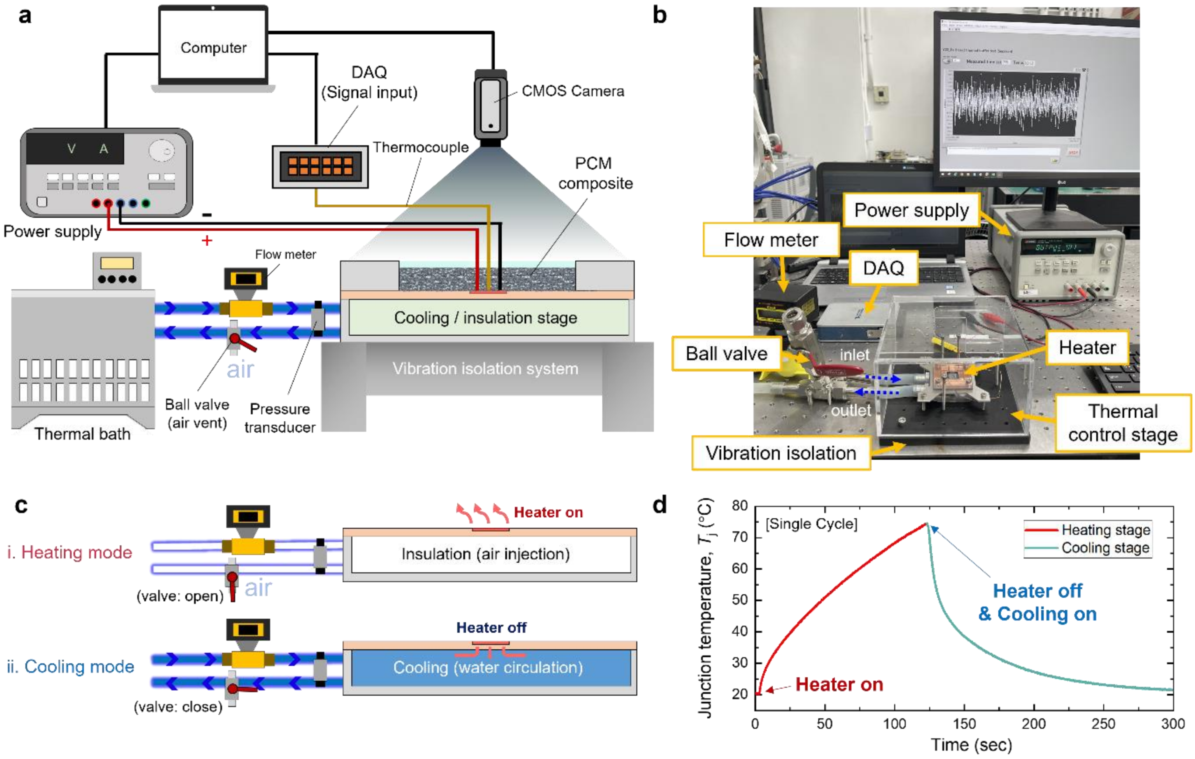

Figure S5. Experimental setup and procedure of thermal regulation module. a) Overall schematic of the test vehicle. b) Photograph of the experimental platform. c) Explanary schematic of heating/cooling mode. d) Transient junction temperature along the heating/cooling period.

6. Numerical results of junction temperature, heat dissipation, and liquid fraction along the time at heating mode for PDMS-protected and bulk Ga-based regulation modules. (Figure S6)

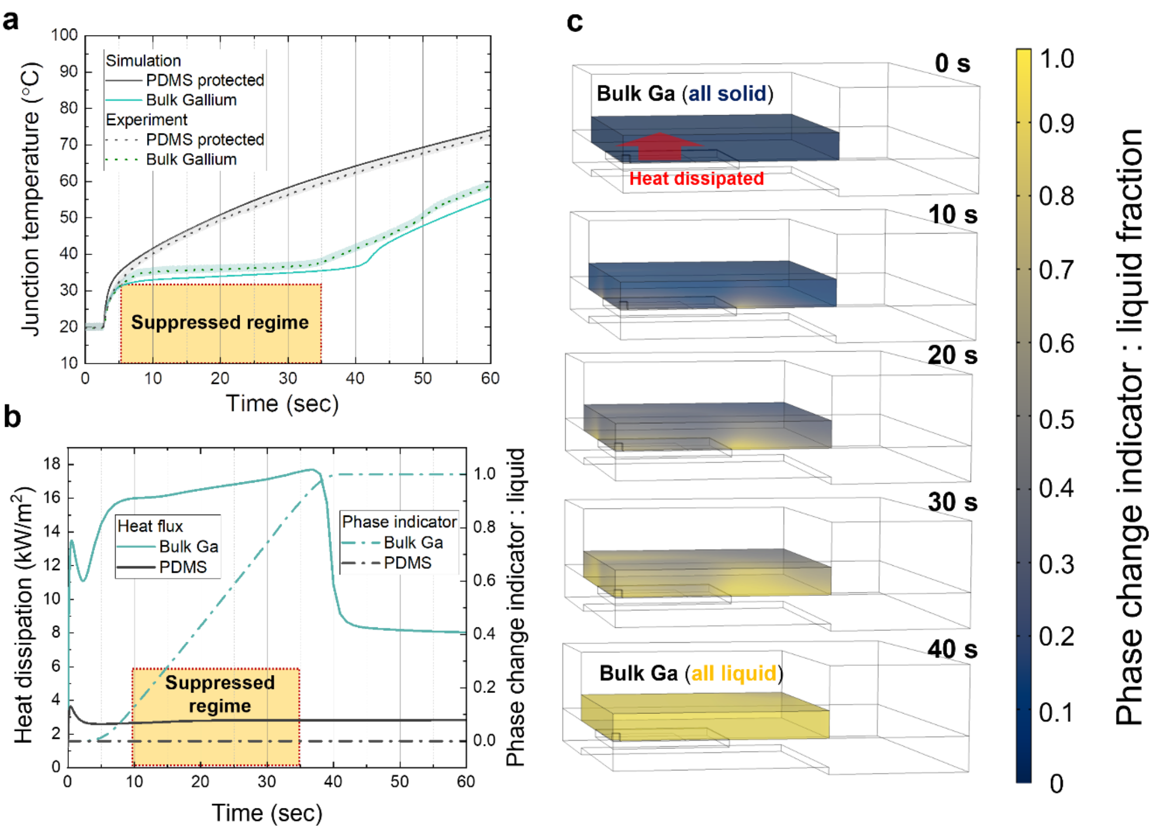

Figure S6. a) Numerical and experimental data of transient junction temperature of microheaters during heating mode (40 W, 1 min). b) Heat dissipation per unit area (kW/m^2) and liquid phase indicator of both modules. c) Color mapping of solid (navy)-liquid (yellow) phase change indicator.

7. Stability of the porous-shaped Ga/PDMS composite over 100 heat-cool cycles (Figure S7)

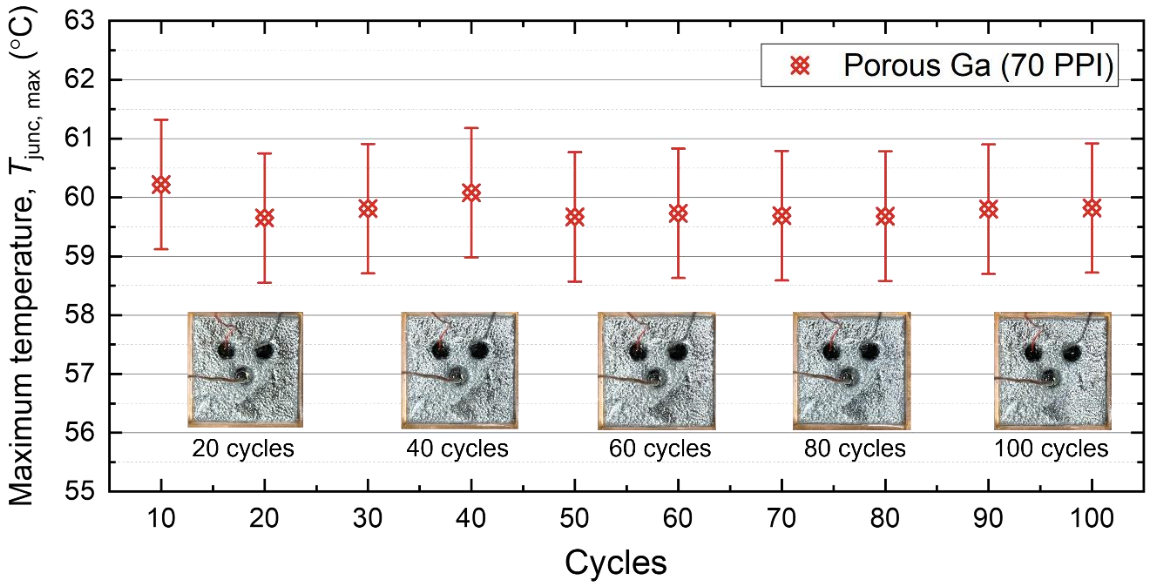

Figure S7. The maximum junction temperature ($T_{junc, max}$) along a hundred cycles of heating-cooling. Inset images provide a fully solidified Ga liquid metal after each cooling mode within the porous-shaped Ga/PDMS composite.

8. Numerical results of temperature and liquid fraction of Ga-PCM along the time (Figure S8)

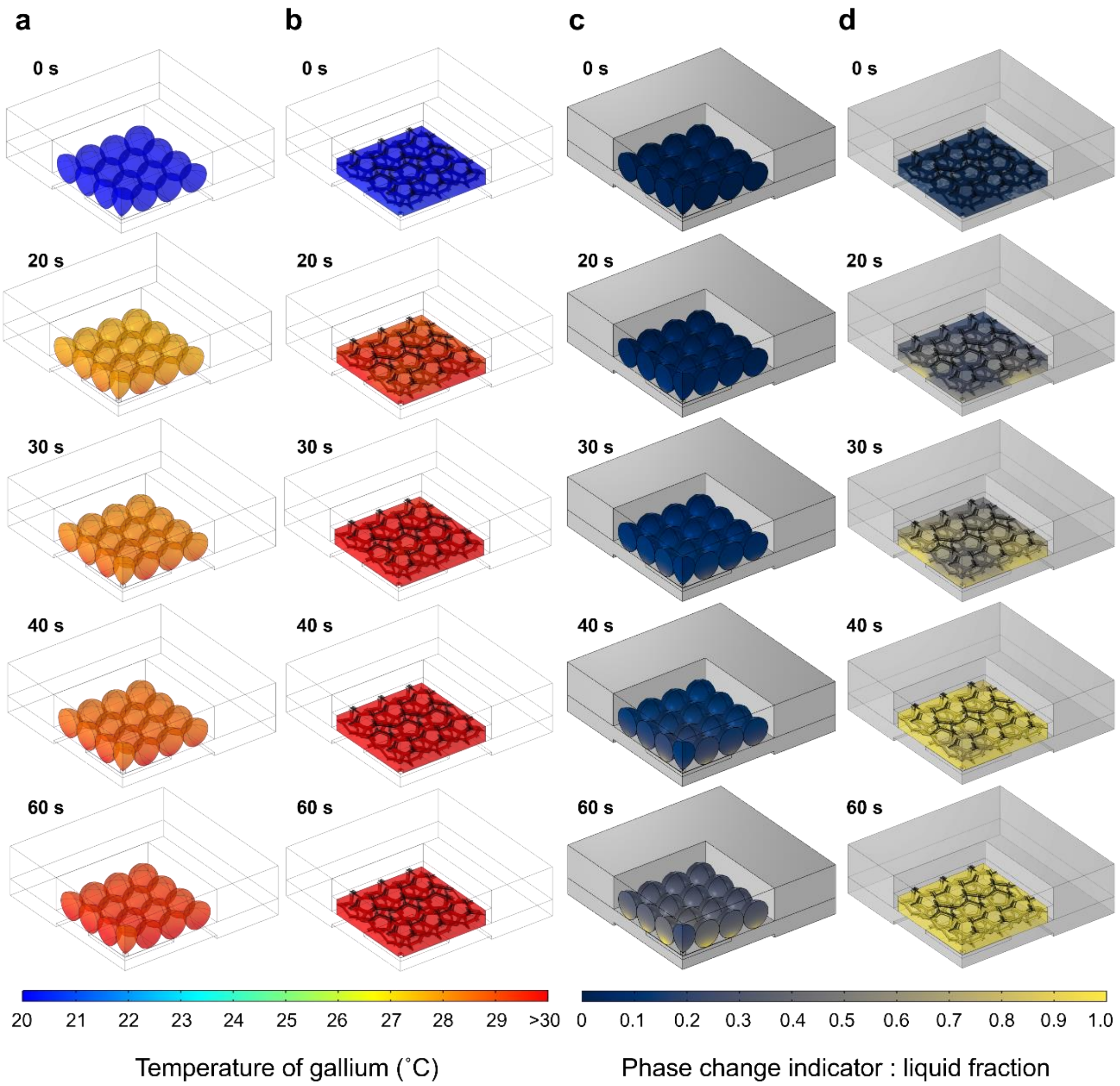

Figure S8. Numerical results of temperature and liquid fraction of Ga-PCM along the time at heating mode. Temperature variation of a) droplet- and b) porous-shaped Ga/PDMS composites. Upper limit of scale bar is set as 30°C to indicate the time for reaching T_{melt} of Ga. Solid (navy) and liquid (yellow) fractions of c) droplet- and d) porous-shaped Ga/PDMS composite.

9. Comparison of specific surface area between droplet- and porous-shaped Ga/PDMS composites (Figure S9)

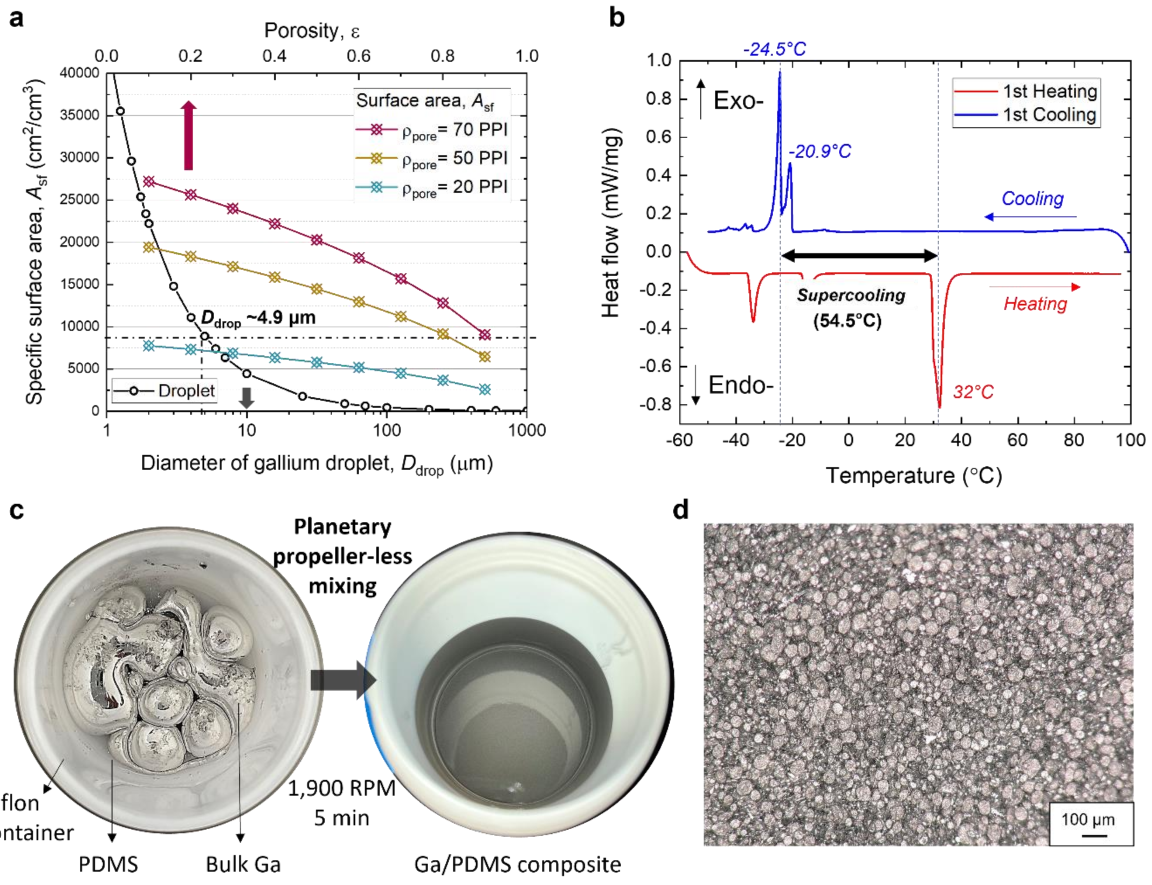

Figure S9. a) Calculated specific surface area of droplet- and porous-shaped Ga/PDMS composite. b) DSC results of fabricated tens of micron-scale Ga/PDMS composite. c) Snapshots of the composite before/after planetary, propeller-less mixing. d) Optical microscope image of the composite.

10. Fabrication process of Pt-based microheaters (Figure S10)

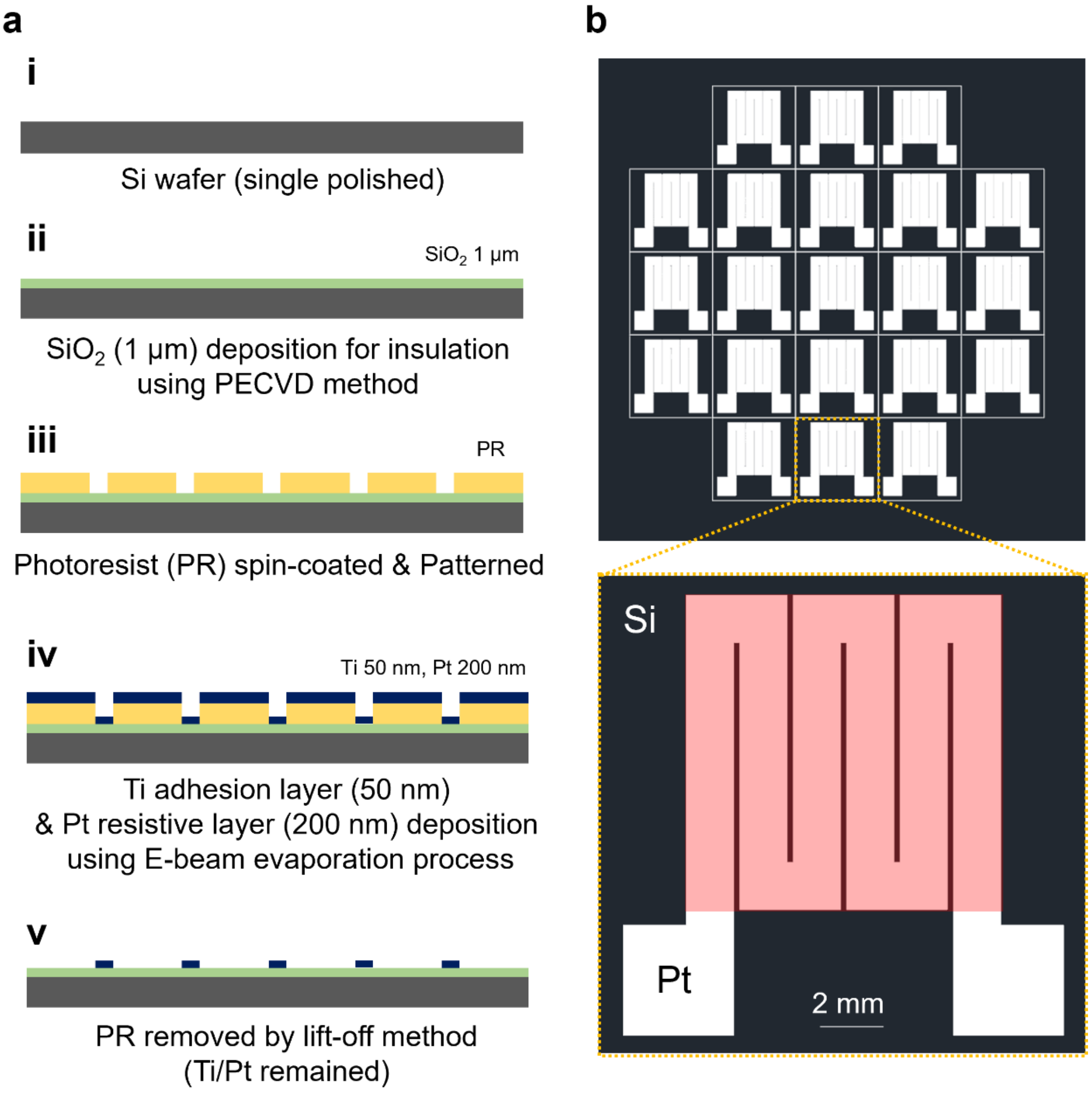

Figure S10. Fabrication process of Pt-based microheaters. a) Fabrication procedure of platinum resistive layer-based microheater. b) Top view of fabricated microheater in Si wafer. The heating area is indicated by red shaded.

11. A quarter-schematic of the simulation model (Figure S11)

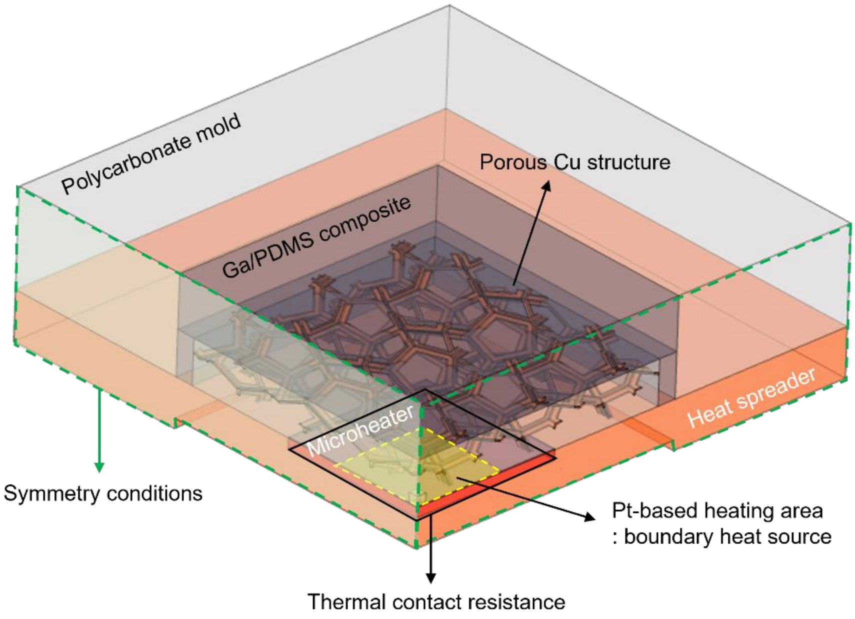

Figure S11. A quarter-schematic of the simulation model. Yellow-shaded area indicate heating area on the microheater (Pt-based pattern). The two areas surrounded by green-dot lines indicate areas for symmetry conditions.

12. Conditions for injection of liquid Ga for various droplet size (Table S1)

Table S1. Conditions for injection of liquid gallium for various droplet size

Syringe tip type	Environment	Outer diameter/Inner diameter of syringe (μm)	Velocity, v_{inj} (mL/min)	Diameter, D_{Ga}
Teflon	Acidic	360 / 100	0.5	Avg. 840 μm
Teflon	Air	813 / 230	0.1	Avg. 2.2 mm
Stainless Steel (SUS)	Air	718 / 413	0.5	Avg. 4 mm

13. Characteristics of the investigated thermal regulation module. (Table S2)

Table S2. Characteristics of the investigated regulation module.

Component	Materials	Dimension (mm^3)	k ($Wm^{-1}K^{-1}$)	C_p ($J g^{-1}K^{-1}$)
Phase change material ; PCM volume	Gallium/PDMS	30 x 30 x 5	PDMS: 0.15<br>Ga_s: 40.6<br>Ga_l: 28.1	PDMS: 1.46<br>Ga_s: 0.37<br>Ga_l: 0.41
Mold for PCM	Polycarbonate	50 x 50 x 5	0.22	1.2
Microheater	Silicon	16 x 16 x 0.5	Func. of T	0.7
Heat spreader	Copper	50 x 50 x 2.5	398	0.385
Temp. control stage	Polycarbonate	50 x 50 x 20	0.22	1.2
Working fluid	Water/Air	Not available	Water: 0.6<br>Air: 0.02	Water: 4.18<br>Air: 1.0
